# The immunomodulatory effects of TNF-α inhibitors on human Th17 cells via RORγt histone acetylation

**DOI:** 10.18632/oncotarget.13791

**Published:** 2016-12-03

**Authors:** Yi-Ching Lin, Yu-Chih Lin, Cheng-Chin Wu, Ming-Yii Huang, Wen-Chan Tsai, Chih-Hsing Hung, Po-Lin Kuo

**Affiliations:** ^1^ Graduate Institute of Clinical Medicine, College of Medicine, Kaohsiung Medical University, Kaohsiung, Taiwan; ^2^ Department of Laboratory Medicine, Kaohsiung Medical University Hospital, Kaohsiung Medical University, Kaohsiung, Taiwan; ^3^ Department of Pediatrics, Kaohsiung Medical University Hospital, Kaohsiung Medical University, Kaohsiung, Taiwan; ^4^ Department of Laboratory Medicine, Faculty of Medicine, College of Medicine, Kaohsiung Medical University, Kaohsiung, Taiwan; ^5^ Division of General Internal Medicine, Department of Internal Medicine, Kaohsiung Medical University Hospital, Kaohsiung Medical University, Kaohsiung, Taiwan; ^6^ Division of Rheumatology, Department of Internal Medicine, Kaohsiung Medical University Hospital, Kaohsiung Medical University, Kaohsiung, Taiwan; ^7^ Department of Radiation Oncology, Kaohsiung Medical University Hospital, Kaohsiung Medical University, Kaohsiung, Taiwan; ^8^ Department of Pediatrics, Kaohsiung Municipal Hsiao-Kang Hospital, Kaohsiung, Taiwan; ^9^ Department of Pediatrics, Faculty of Medicine, College of Medicine, Kaohsiung Medical University, Kaohsiung, Taiwan; ^10^ Research Center for Environmental Medicine, Kaohsiung Medical University, Kaohsiung, Taiwan; ^11^ Institute of Medical Science and Technology, National Sun Yat-Sen University, Kaohsiung, Taiwan

**Keywords:** TNF-α, Th17, RORγt, histone acetylation, rheumatoid arthritis

## Abstract

The presence of interleukin (IL)-17-related cytokines correlates with rheumatoid arthritis (RA) pathogenesis. Epigenetic modifications, including histone acetylation, regulate gene expression in RA pathogenesis. Tumour necrosis factor-alpha (TNF-α) inhibitors such as etanercept and adalimumab, represent a breakthrough in RA treatment. We aimed to investigate the effects of etanercept and adalimumab on human Th17-polarized cells and the possible intracellular regulators of these effects, including the Th17-specific transcription factors signal transducer, activator of transcription 3 (STAT3), retinoid-related orphan receptor γ-T (RORγt) and epigenetic modification. Human CD4^+^ T cells from healthy subjects and patients with RA were pretreated with TNF-α inhibitors and then being polarized into IL-17-producing cells. The Th17-related cytokine levels in the culture supernatants were determined with an enzyme-linked immunosorbent assay. Intracellular signalling was investigated by western blot, real-time RT-PCR, and chromatin immunoprecipitation. Th17-polarized cells from patients with RA produced more IL-17A, IL-17F and IL-22 than those from healthy subjects. Etanercept and adalimumab suppressed IL-17A, IL-17F and IL-22 levels in Th17-polarized cells from healthy subjects and patients with RA. Western blot analysis revealed that etanercept and adalimumab decreased mitogen-activated protein kinase-phospho-p38, nuclear factor-κB-phospho-p65, phospho-STAT3 and RORγt levels. Etanercept and adalimumab decreased histone (H)3 and H4 acetylation in the RORγt gene promotor region by decreasing the recruitment of the acetyltransferases p300, CBP and PCAF. The present study broadens our knowledge of the mechanisms underlying the immunomodulatory effects of TNF-α inhibitors in rheumatoid arthritis treatment.

## INTRODUCTION

Rheumatoid arthritis (RA), characterized by joint inflammation, synovial hyperplasia and excessive bone resorption, is mediated by many immune cells and inflammatory cytokines. T helper (Th) 17 cells, a distinct lineage of Th cells, produce interleukin (IL)-17 and play an important role in RA [1]. The frequency of Th17 cells is reportedly higher in peripheral blood mononuclear cells from patients with RA than healthy subjects [2]. Higher IL-17 levels were found to be produced by RA synovium but not osteoarthritis, and these high IL-17 levels were positively correlated with the severity of the disease [3, 4].

TNF-α inhibitors, which are biologic agents blocking TNF-α, represent a breakthrough in the treatment of RA [5]. Etanercept (Enbrel™) is a recombinant protein consisting of the human TNF receptor coupled with the Fc portion of human IgG. Adalimumab (Humira™) is a human anti-human TNF-α antibody. Both etanercept and adalimumab have been approved by the U.S. Food and Drug Administration (FDA) for human use to prevent inflammatory processes in RA. In RA patients treated with TNF-α inhibitors, serum Th17-related cytokines decreased significantly in parallel with clinical remission in the responders, whereas increased percentage of Th17 cell and elevating related cytokine levels were found in non-responders [6, 7]. However, mechanisms of how these TNF-α inhibitors suppress cytokine production, especially the immunomodulatory effects on Th17 cells, are yet to be elucidated.

The mitogen-activated protein kinase (MAPK) and nuclear factor-κB (NFκB) pathways, which could be activated by TNF receptor, have been found to affect cytokine gene expression that are associated with the pathogenesis of RA [8, 9]. MAPKs, including ERK, p38 and JNK, are considered the possible therapeutic targets for RA because they regulate proinflammatory cytokine production and play important roles in the signalling cascades [10, 11]. NFκB is central to the production of proinflammatory mediators in the inflamed synovium of RA. Once activated, the ability of NFκB to induce transcription can further be enhanced by post-translational phosphorylation and acetylation [12, 13]. Signal transducer and activator of transcription 3 (STAT3) and retinoic acid-related orphan receptor gamma t (RORγt) are linage-specific transcription factors that function in Th17 differentiation [14, 15]. In rheumatoid synovial T cells, modulation of STAT3 suppresses Th17 differentiation and increases the proportion of regulatory T cells [16]. It has been reported that RORγt-specific transcriptional inhibition suppresses autoimmunity associated with Th17 cells [17]. Because MAPKs, NFκB, STAT3 and RORγt are key regulators in RA pathogenesis and Th17 differentiation, we investigated whether etanercept and adalimumab would exert immunomodulatory effects on Th17-polarized cells by regulating these mediators.

Epigenetics refers to heritable changes in gene function that do not involve changes in nucleotide sequence [18]. Epigenetic changes can result in gene dysregulation, causing various pathological conditions, including autoimmune diseases. Recently, efforts to understand the non-genetic contributions to RA susceptibility have focused on investigating epigenetic mechanisms [19, 20]. There are increasing studies about the role of epigenetics in DNA methylation in RA synovial fibroblasts or peripheral blood mononuclear cells (PBMCs) but less about histone modifications [21]. It has been found that the balance between histone acetyltransferase (HAT) and histone deacetylase (HDAC) activity is strongly shifted towards histone acetylation in RA synovial tissue [22, 23]. The roles of TNF-α inhibitors in the epigenetic modifications underlying Th17 differentiation and their functions are still unclear.

In the present study, we examined the effects of etanercept and adalimumab on Th17-related cytokines in Th17-polarized cells from healthy subjects and patients with RA. We further investigated the possible intracellular regulators, including epigenetic modification. Understanding how Th17 cells and their downstream cytokines act at a fundamental level is likely to reveal new strategies for treating RA.

## RESULTS

### Induction of IL-17A and IL-17F expression by human CD4^+^ T cells in Th17-polarized conditions

**Figure 1:**
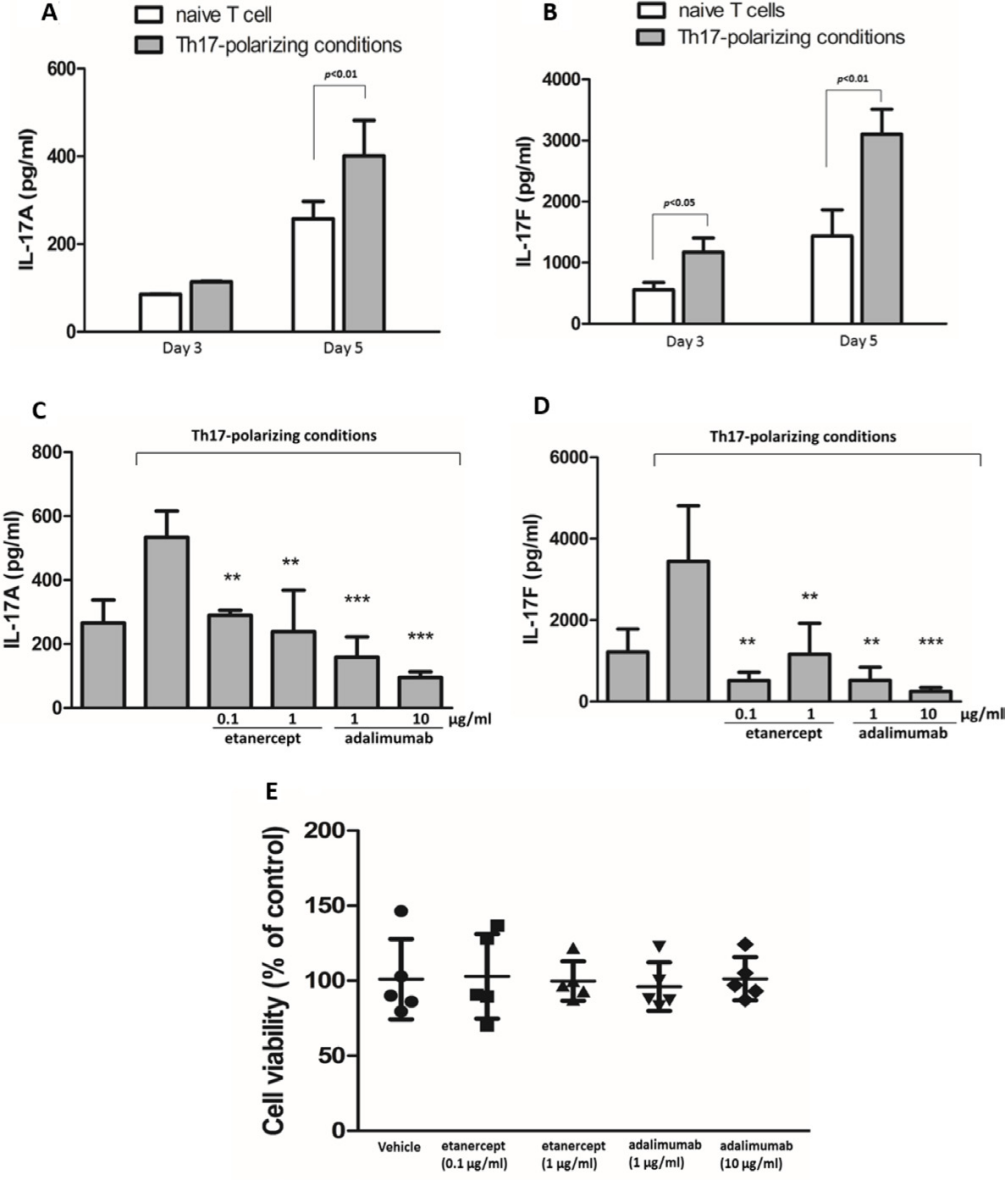
Etanercept and adalimumab suppress IL-17A and IL-17F production in human Th17-polarized T cells Human naïve CD4^+^ T cells polarized towards the Th17 phenotype in the presence of rhIL-2, rhIL-1β, rhIL-23, rhTGF- β and rhIL-6 with anti-hCD3, anti-hCD28, anti-hIL-4, and anti-hINF-γ, and then were cultured for 3 days or 5 days. Control naïve T cells were cultured only in the presence of anti-hCD3, anti-hCD28 and rhIL-2. The supernatants were collected for (**A**) IL-17A and (**B**) IL-17F detection by ELISA. The results represent the means ± standard deviations of three independent experiments. Pretreatment with etanercept (0.1 and 1 μg/ml) or adalimumab (1 and 10 μg/ml) suppressed (**C**) IL-17A and (**D**) IL-17F production in human CD4^+^ T cells from a healthy individual after 5 days of Th17-polarized conditions. The results represent the means ± standard deviations of three experiments. *P < 0.05, **P < 0.01 and ***P < 0.001 between the Th17-polarized conditions with and without TNF-α inhibitor pretreatment. (**E**) The viability of human CD4^+^ T cells pretreated with or without etanercept (0.1 and 1 μg/ml) or adalimumab (1 and 10 μg/ml) was determined after 5 days of Th17 polarization using the WST-1 assay and expressed as a percentage of the control. The results represent the means ± standard deviations of 5 individual experiments.

Th17 cells produce specific cytokines, including IL-17A and IL-17F. In the present study, we successfully established Th17-polarized conditions by culturing purified CD4^+^ T cells from human subjects in the presence of rhIL-2, rhIL-1β, rhIL-23, rhTGF-β and rhIL-6 with anti-CD3 antibody, anti-CD28 antibody, anti-hIL-4 antibody and anti-hIFN-γ antibody. Compared with conventional CD4^+^ T cells that were co-cultured with anti-CD3 antibody, anti-CD28 antibody and rhIL-2, Th17-polarized cells produced significant amounts of both IL-17A and IL-17F after 5 days of culture, as determined by ELISA analysis (Figures 1A and 1B).

### Etanercept and adalimumab suppress IL-17A and IL-17F expression in human Th17-polarized cells

Th17-polarized human CD4^+^ T cells were pretreated with etanercept at 1 and 0.1 μg/mL or adalimumab at 1 and 10 μg/mL for 2 h prior to Th17 polarization. The results revealed that both IL-17A and IL-17F expression in the Th17-polarized cells was significantly suppressed by etanercept (0.1 and 1 μg/mL) and adalimumab (1 and 10 μg/mL) after 5 days of Th17 polarization (Figure 1C and 1D). Following observation of the suppressive effects of etanercept and adalimumab on IL-17A and IL-17F expression in Th17-polarized cells, we determined the cytotoxic effects of the different concentrations of etanercept and adalimumab using a WST-1 cell viability assay. As illustrated in Figure 1E, neither etanercept (0.1 and 1 μg/mL) nor adalimumab (1 and 10 μg/mL) significantly reduced the viability of the Th17-polarized cells compared with vehicle after 5 days of incubation. This result suggested that etanercept and adalimumab exert no cytotoxic effects on Th17-polarized cells.

### The effects of etanercept and adalimumab on IL-17A, IL-17F and IL-22 levels in Th17-polarized cells from patients with RA

**Figure 2:**
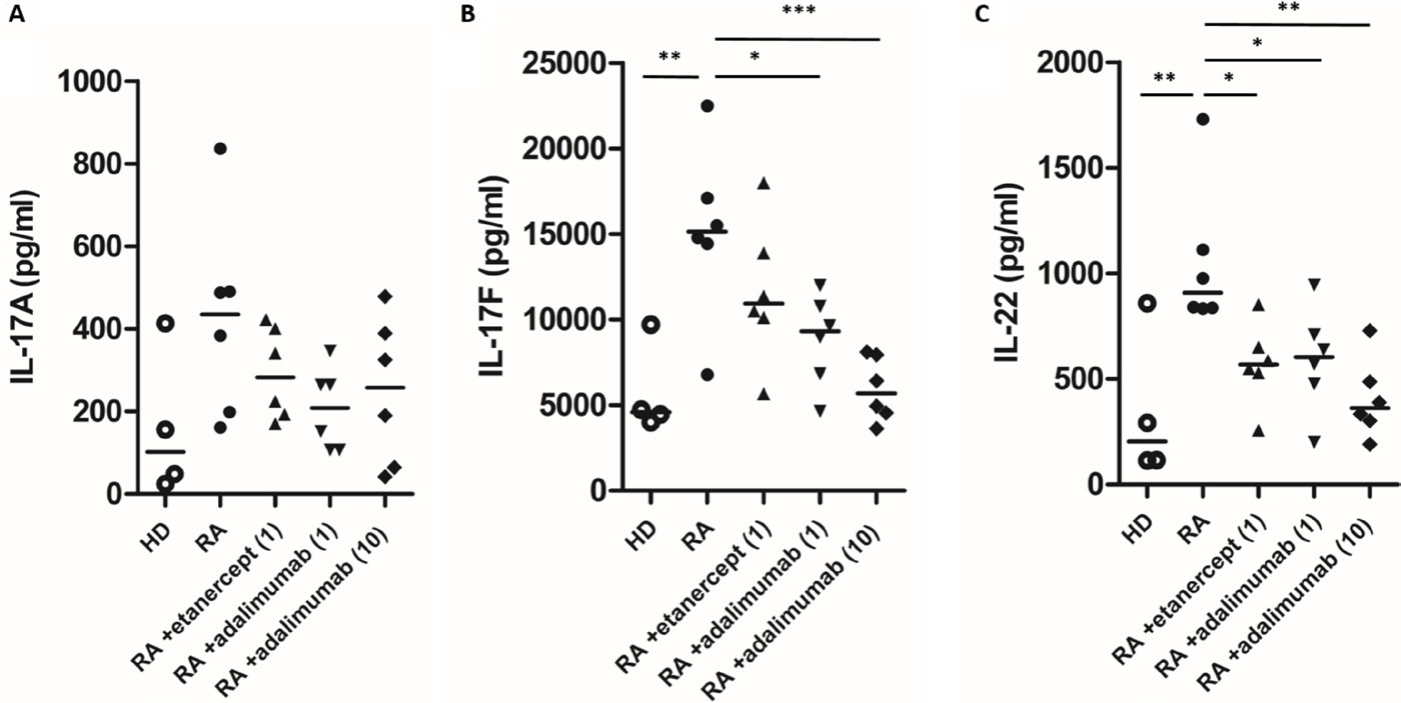
The effects of etanercept and adalimumab on IL-17A, IL-17F and IL-22 production in Th17-polarized cells from patients with RA The levels of Th17-related cytokines, including (**A**) IL-17A, (**B**) IL-17F and (**C**) IL-22, in the supernatants of Th17-polarized cells from four healthy donors (HD) and six patients with RA that were pretreated *in vitro* with or without etanercept (1 μg/ml) or adalimumab (1 or 10 μg/ml) were determined by ELISA. Horizontal bars indicate the median. *P < 0.05, **P < 0.01 and ***P < 0.001.

We also tested the effects of etanercept and adalimumab on Th17-polarized cells from patients with RA. Supernatants were collected from Th17-polarized cells from six patients with RA with or without etanercept (1 μg/mL) or adalimumab (1 and 10 μg/mL) pretreatment *in vitro*. As illustrated in Figure 2, the supernatants of Th17-polarized cells from patients with RA contained significant amounts of IL-17A, IL-17F and IL-22, and there were statistically significant differences in IL-17F and IL-22 levels compared with cells from healthy subjects. In the RA group, IL-17F expression was significantly suppressed by adalimumab at 1 and 10 μg/mL, and IL-22 levels were significantly suppressed by *in vitro* pretreatment with etanercept at 1 μg/mL or adalimumab at 1 or 10 μg/mL (Figure 2A, and 2C). Th17-polarized cells from patients with RA produced more amount of IL-17A, IL-17F and IL-22 than cells from healthy subjects, and the suppressive effects of TNF-α inhibitors predominantly affected the expression of IL-17F and IL-22.

### Etanercept and adalimumab suppress IL-17A, IL-17F and IL-22 production in human Th17-polarized cells through MAPK pathways

**Figure 3:**
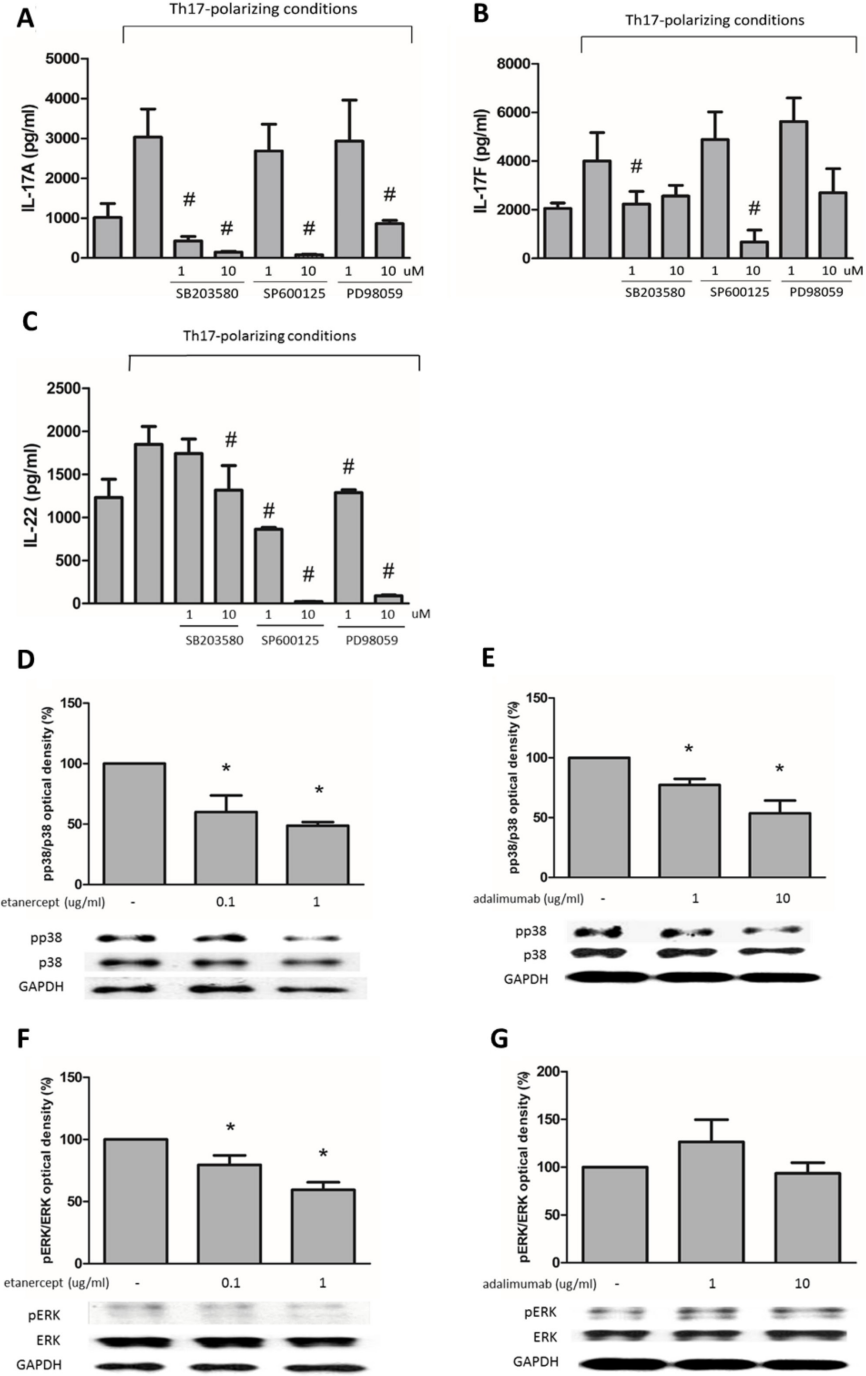
The suppressive effects of etanercept and adalimumab on IL-17A, IL-17F and IL-22 expression in human Th17-polarized cells through MAPK pathways (**A**) Human Th17-polarized cells were induced from purified CD4^+^ T cells from healthy subjects. Pretreatment with SB203580 (a p38 inhibitor, 10^−6^–10^−5^ M), SP600125 (a JNK inhibitor, 10^-5^ M) or PD98059 (an ERK inhibitor, 10^−5^ M) significantly suppressed IL-17A expression in Th17-polarized cells. (**B**) SB203580 (10^-6^ M) and SP600125 (10^−5^ M) significantly suppressed IL-17F expression in human Th17-polarized cells. (**C**) Pretreatment with SB203580 (10^−5^ M), SP600125 (10^−6^–10^−5^ M) and PD98059 (10^−6^–10^−5^ M) could significantly suppress IL-22 expression in Th17-polarized cells. ^#^*P* < 0.05 for the comparison of Th17-polarized conditions with and without MAPK inhibitor pretreatment. (**D**) Etanercept (0.1 and 1 μg/ml) and (**E**) adalimumab (1 and 10 μg/ml) decreased pp38 levels in human Th17-polarized cells. (**F**) Etanercept (0.1 and 1 μg/ml) but not (**G**) adalimumab decreased pERK levels in human Th17-polarized cells. For western blot analysis, the standard deviations of the optical density data were calculated for three independent experiments, and one experiment representative of the set of three is shown. *P < 0.05 for the comparison of Th17-polarized conditions with and without etanercept or adalimumab pretreatment.

IL-17A expression was suppressed by SB203580 (a p38 inhibitor, 10^-6^–10^-5^ M), SP600125 (a Jun NH2-terminal kinase (JNK) inhibitor, 10^−5^ M) and PD98059 (an extracellular signal–related kinase (ERK) inhibitor, 10^−5^ M) (Figure 3A). IL-17F expression was suppressed by SB203580 (10^−6^ M) and SP600125 (10^−5^ M) but not PD98059 (10^−6^–10^−5^ M) (Figure 3B). IL-22 expression was suppressed by SB203580 (10^−5^ M), SP600125 (10^−6^–10^−5^ M) and PD98059 (10^−6^–10^−5^ M) (Figure 3C). In western blot analysis, phospho-p38 (p38) expression was significantly suppressed by etanercept at 0.1 and 1 μg/mL and adalimumab at 1 and 10 μg/mL (Figure 3D and 3E), and phospho-ERK (pERK) expression was suppressed by etanercept at 0.1 and 1 μg/mL (Figure 3F) but not by adalimumab (Figure 3G). Western blot analysis indicated that etanercept and adalimumab did not significantly suppress phospho-JNK (pJNK) expression in Th17-polarized cells by (data not shown). These results suggested that the suppression of IL-17A, IL-17F and IL-22 production by etanercept and adalimumab occurs through MAPK pathways in Th17-polarized cells.

### Etanercept and adalimumab decrease phosphorylation of NFκB and STAT3 in human Th17-polarized T cells

**Figure 4:**
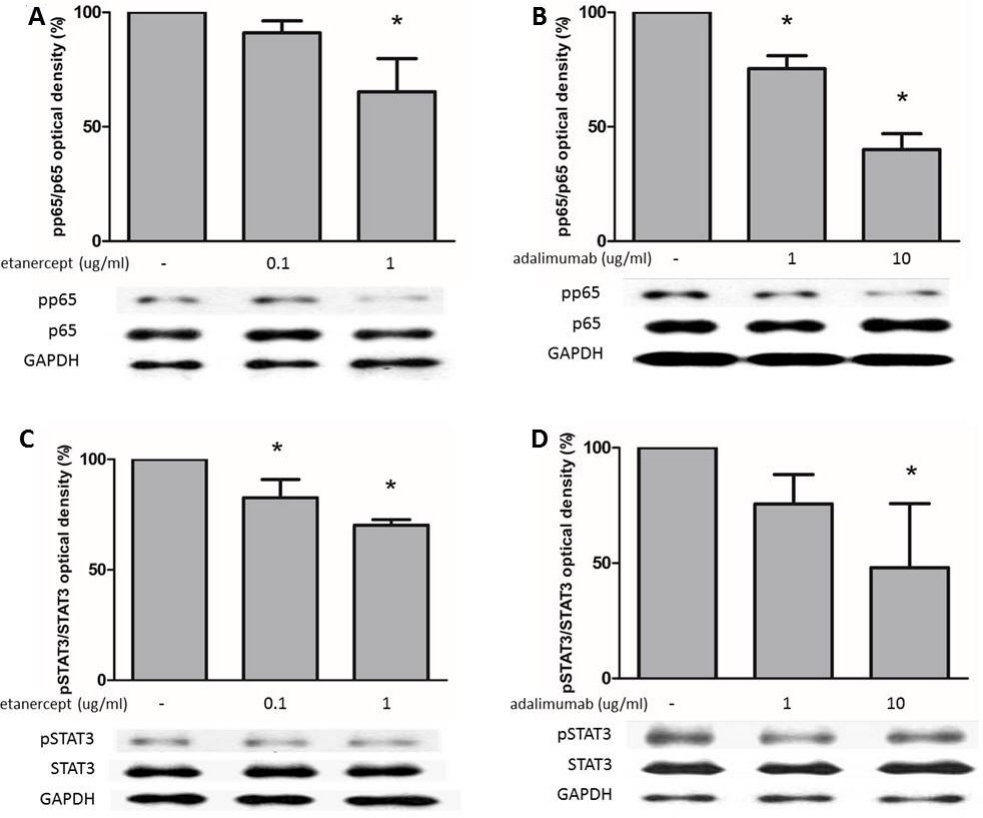
Etanercept and adalimumab downregulate NFκB and STAT3 expression in human Th17-polarized cells (**A**) Th17-polarized cells were induced from purified CD4^+^ T cells from healthy subjects. Etanercept at 1 μg/ml and (**B**) adalimumab at 1 and 10 μg/ml decreased pp65 levels in human Th17-polarized cells. (**C**) Etanercept at 0.1 and 1 μg/ml and (**D**) adalimumab at 10 μg/ml decreased pSTAT3 levels in human Th17-polarized cells. For western blot analysis, the standard deviations of the optical density data were calculated for three independent experiments, and one experiment representative of the set of three is shown. *P < 0.05 for the comparison of Th17-polarized conditions with or without etanercept or adalimumab pretreatment.

The NFκB pathway plays a key role in regulating the immune response. STAT3 is an important transcription factor in Th17-cell differentiation [24]. As shown in Figure 4, western blot analysis revealed that phospho-p65 (pp65)-NFκB levels were significantly suppressed by etanercept at 1 μg/mL (Figure 4A) and adalimumab at 1 and 10 μg/mL (Figure 4B). Phospho-STAT3 (pSTAT3) expression was significantly suppressed by etanercept at 0.1 and 1 μg/mL (Figure 4C) and adalimumab at 10 μg/mL (Figure 4D). These data suggested that etanercept and adalimumab decreased p65-NFκB and STAT3 phosphorylation in Th17-polarized cells.

### Histone acetylation is involved in the effects of etanercept and adalimumab on RORγt expression in Th17-polarized cells

**Figure 5:**
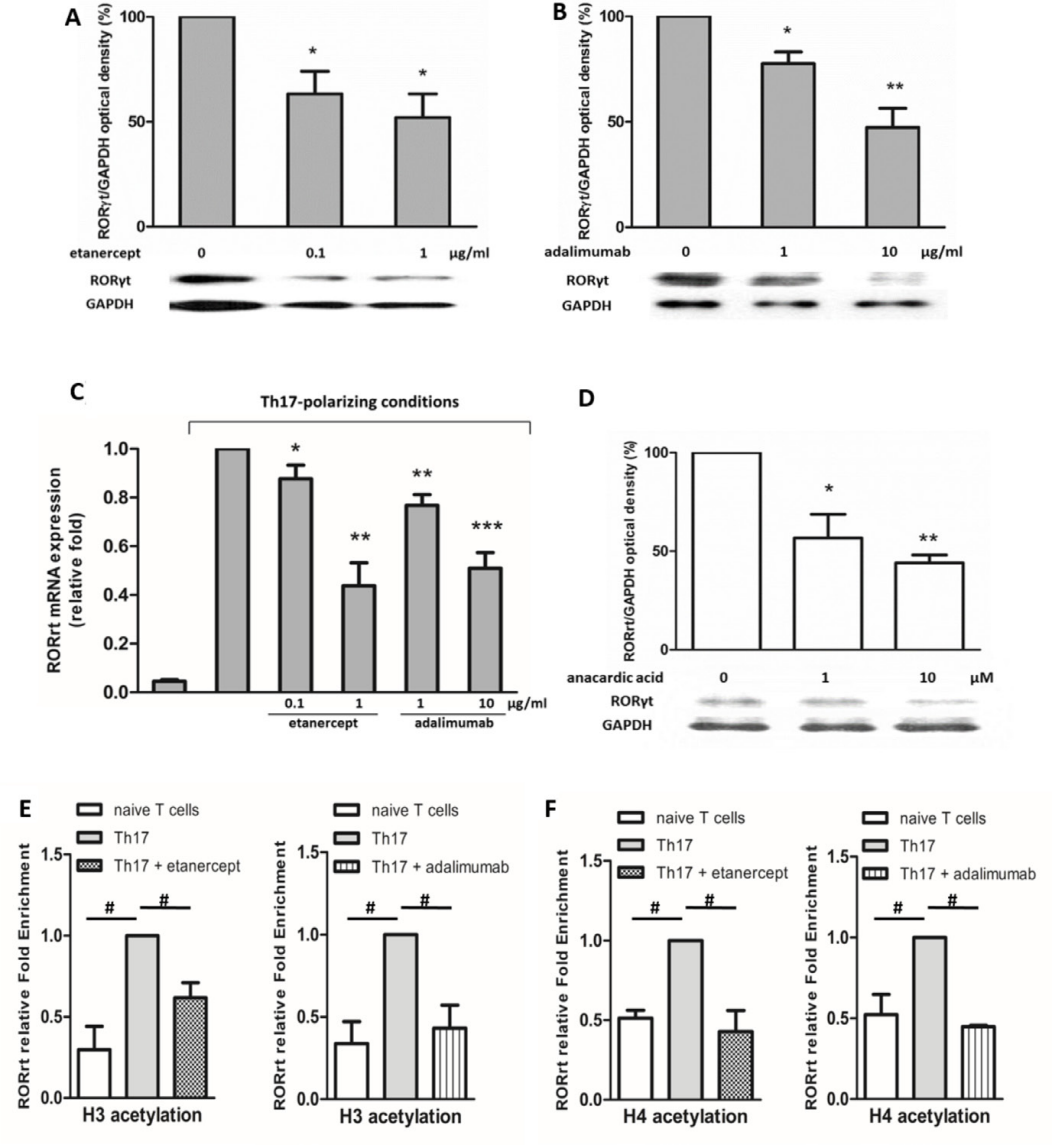
Etanercept and adalimumab downregulate RORγt expression in human Th17-polarized T cells via histone acetylation Western blot analysis revealed that (**A**) etanercept (0.1 and 1 μg/ml) and (**B**) adalimumab (1 and 10 μg/ml) suppressed RORγt expression 24 h after Th17 polarization. (**C**) Real-time quantitative PCR was used to evaluate RORγt mRNA expression in CD4^+^ T cells after the cells were cultured under Th17-polarized conditions for 12 h with or without pretreatment with TNF-α inhibitors for 2 h. The results represent the means ± standard deviations of three independent experiments. (**D**) Pretreatment with anacardic acid (an acetyltransferase inhibitor) suppressed RORγt expression 24 h after Th17 polarization. For western blot analysis, the standard deviations of the optical density data were calculated from three independent experiments, and one experiment representative of the set of three is shown. *P < 0.05, **P < 0.01 and ***P < 0.001 between Th17-polarized conditions with and without TNF-α inhibitor pretreatment. ChIP analysis revealed that histone (**E**) H3 and (**F**) H4 acetylation was decreased by etanercept (1 μg/mL) and adalimumab (10 μg/mL) 6 h after Th17-polarization. The standard deviations of the ChIP data were calculated from four independent experiments. ^#^*P* < 0.05.

**Figure 6:**
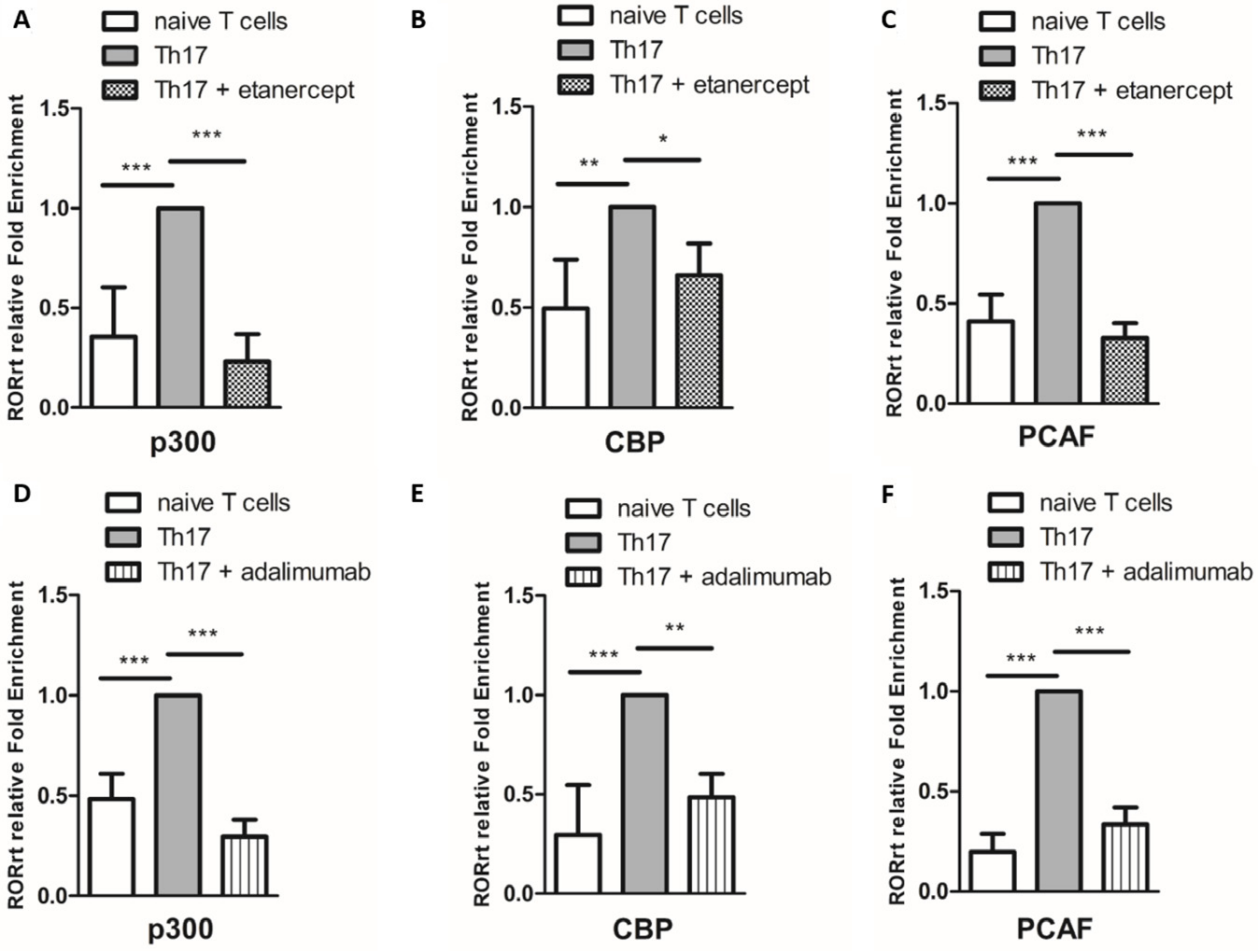
The effects of etanercept and adalimumab on NFκB-associated acetyltransferases in Th17-polarized cells Etanercept (1 μg/mL) decreased the recruitment of the NFκB-associated acetyltransferases (**A**) p300, (**B**) CBP and (**C**) PCAF in Th17-polarized cells induced from purified CD4^+^ T cells (naïve T cells). Adalimumab (10 μg/mL) decreased the recruitment of the NFκB-associated acetyltransferases (**D**) p300, (**E**) CBP and (**F**) PCAF in Th17-polarized cells. The standard deviations of the ChIP data were calculated from four independent experiments. *P < 0.05, **P < 0.01 and ***P < 0.001.

Figure 5 shows that etanercept at 0.1 and 1 μg/mL and adalimumab at 1 and 10 μg/mL suppressed RORγt protein (Figures 5A and 5B) and mRNA (Figure 5C) expression in Th17-polarized cells. Epigenetic modifications are believed to regulate gene expression in RA pathogenesis, so we further examined whether the suppressive effects of etanercept and adalimumab on RORγt expression were mediated by epigenetic regulation. As demonstrated in Figure 5D, pretreatment with anacardic acid (a HAT inhibitor) suppressed RORγt expression in Th17-polarized cells. This finding suggested that histone acetylation may be involved in RORγt expression. A chromatin immunoprecipitation (ChIP) assay revealed that etanercept and adalimumab significantly downregulated H3 and H4 acetylation in the RORγt promoter area (Figures 5E and 5F). These data suggested that TNF-α inhibitors may suppress RORγt expression by inhibiting the acetylation of histones H3 and H4 in Th17-polarized cells. Moreover, because etanercept and adalimumab suppressed NFκB activation, as shown in Figure 4, we used a ChIP assay to verify whether the NFκB-associated acetyltransferases p300, CBP, and PCAF were involved in histone acetylation. As illustrated in Figure 6, both etanercept and adalimumab significantly decreased the recruitment of p300, CREB-binding protein (CBP) and p300/CBP-associated factor (PCAF) to the RORγt promoter area. These data suggested that TNF-α inhibitors suppress RORγt expression by inhibiting the acetylation of histones H3 and H4 by decreasing the recruitment of the histone acetyltransferases p300, CBP and PCAF.

### The *in vitro* effects of etanercept and adalimumab on Th17-related cytokine production in Th17-polarized cells from one RA patient undergoing Enbrel^TM^ treatment

Th17-polarized cells from one RA patient treated with weekly subcutaneous injections of 25 mg Enbrel^TM^ were collected 2 h before and 48 h after Enbrel^TM^ injection. According to the prescribing information [25], the means ± standard deviations of the half-life and the time to the maximum serum concentration (C_max_) in patients with RA after subcutaneous injection of 25 mg of Enbrel™ are 102 ± 30 h and 69 ± 34 h, respectively. Thus, we presumed that the concentration of Enbrel™ in the patient reached the minimum serum concentration (C_min_) 2 h before subcutaneous injection and reached C_max_ 48 h after subcutaneous injection.

**Figure 7:**
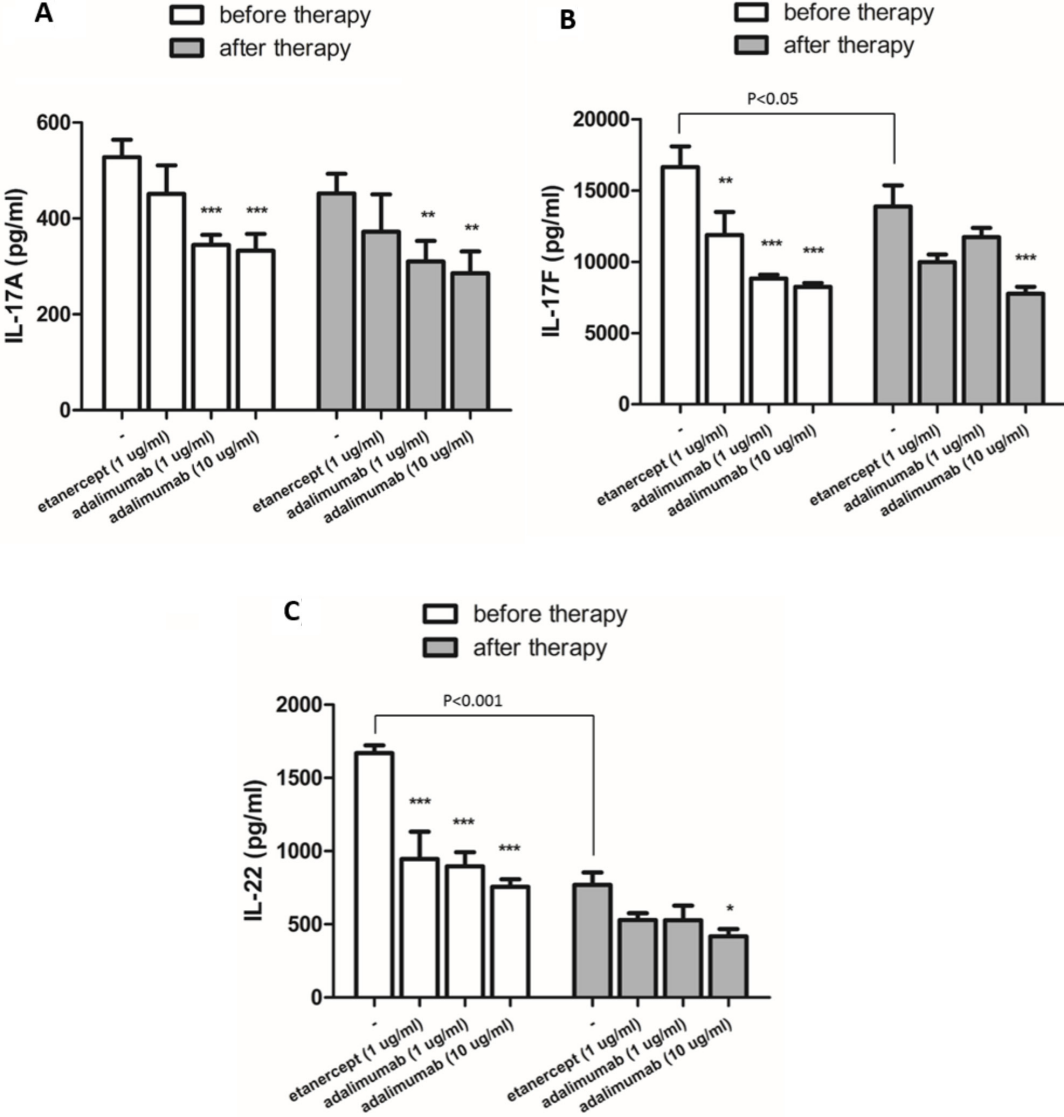
The in vitro effects of etanercept and adalimumab on Th17-related cytokine production by Th17-polarized cells from an RA patient undergoing Enbrel™ treatment Th17-polarized cells induced from CD4^+^ T cells that were collected from an RA patient 2 h before (at the trough level) and 48 h after (at the peak level) of Enbrel™ injection. The *in vitro* effects of etanercept (1 μg/mL) and adalimumab (1 and 10 μg/mL) on IL-17A, IL-17F and IL-22 production in Th17-polarized cells were determined by ELISA. *P*-values indicate comparison of the cytokine levels before and after Enbrel™ injection. (**A**) IL-17A production by Th17-polarized cells isolated from the RA patient 2 h before and 48 h after Enbrel™ injection was significantly suppressed by *in vitro* treatment with adalimumab (1 and 10 μg/mL) but not etanercept (1 μg/mL). (**B**) IL-17F and (**C**) IL-22 production was significantly suppressed by *in vitro* treatment with etanercept (1 μg/mL) and adalimumab (1 μg/mL) when cells were isolated from the RA patient 2 h before but not 48 h after Enbrel™ injection. *In vitro* pretreatment with adalimumab (10 μg/mL) significantly suppressed IL-17F and IL-22 production both before and after Enbrel™ injection. The bars represent the means ± standard deviations from three independent experiments. *P < 0.05, **P < 0.01 and ***P < 0.001 between the Th17-polarized cells with and without *in vitro* etanercept or adalimumab pretreatment.

As illustrated in Figure 7, the supernatants of Th17-polarized cells collected at the Enbrel™ C_min_ (shown as “before therapy”) contained significant levels of IL-17A, IL-17F and IL-22, and these IL-17F and IL-22 levels were significantly different from the levels in the supernatants collected at the Enbrel™ C_max_ (shown as “after therapy”). As shown in Figure 7A, IL-17A expression was not significantly suppressed by *in vitro* etanercept (1 μg/mL) in Th17-polarized cells collected at either the C_max_ or the C_min_ of Enbrel™. Adalimumab at both 1 μg/mL and 10 μg/mL significantly suppressed IL-17A expression in Th17-polarized cells collected at both the C_max_ and the C_min_ of Enbrel™. IL-17F and IL-22 levels both were significantly suppressed by *in vitro* etanercept (1 μg/mL) and adalimumab (1 and 10 μg/mL) in Th17-polarized cells collected at the C_min_ of Enbrel™, but only adalimumab at 10 μg/mL produced significant suppression on these cytokines when cells were collected at the C_max_ of Enbrel™ (Figure 7B and 7C). These results suggest that the ability of Th17-polarized cells to produce cytokines decreased when the patient was treated with higher concentrations of anti-TNF-α therapy. Also, the suppressive effects of a different TNF-α inhibitor on *in vitro* cytokine production by Th17-polarized cells from the patient treated with anti-TNF-α therapy seemed to be similar when the inhibitors were used at the same concentration.

## DISCUSSION

The population of Th17 cells is small under physiologic conditions, and factors inducing Th17 cell differentiation in humans are more obscure than those in animal models of arthritis. In the present study, we successfully polarized human CD4^+^ T cells to produce significant amounts of IL-17A and IL-17F after 5 days of incubation with recombinant TGF-β, IL-6, IL-1β and IL-23. We demonstrated that IL-17A and IL-17F production by normal Th17-polarized cells were significantly suppressed by both etanercept and adalimumab. The suppressive effects of etanercept and adalimumab were also found in Th17-polarized cells from patients with RA. IL-17A, IL-17F and IL-22 levels were higher in the supernatants of Th17-polarized cells generated from the CD4^+^ T cells of patients with RA than the cells generated from CD4^+^ T cells of healthy subjects. This suggests that the CD4^+^ T cells of patients with RA may be more sensitive to Th17 polarization and the associated cytokine production. Moreover, in a patient already receiving regular etanercept treatment, we found that the *in vitro* suppressive effects of the same concentrations (1 μg/mL) of etanercept and adalimumab were similar, and the suppressive effects of adalimumab on Th17-related cytokine production were significantly greater at a higher therapeutic concentration (10 μg/mL). It has been reported that there are associations between adalimumab serum levels and therapeutic response in patients with RA [26]. Baseline adalimumab levels were significantly higher in patients with persistent remission (median 10.5 μg/mL) than those with disease flare-up (median 0.9 μg/mL). In our study, the suppressive effects of adalimumab in patients with RA were dose dependent, especially for IL-17F and IL-22 production. These results were in line with clinical observations and might provide further understanding of the therapeutic effects of anti-TNF-α therapy.

It has been reported that the TNF receptor facilitates the activation of MAPK kinases (MAPKK) and NFκB (IκB) kinases and subsequently phosphorylates MAPK and NFκB [8]. In the present study, TNF-α inhibitors suppressed pp38-MAPK, pERK-MAPK, pp65-NFκB and phospho-STAT3 expression. MAPKs, including p38, ERK and JNK, mediate cytokine production and are part of the signalling cascades downstream of the T cell receptor (TCR), toll-like receptors (TLRs) and IL-1 and TNF-α receptors [11]. Growth factors, stress stimuli or cytokines can phosphorylate and activate MAPKs, which activate downstream transcription factors such as Elk-1, c-Myc, activator protein 1 (AP-1), activating transcription factor (ATF)-2 and MAPK-activated protein-2 by activating MAPK kinase kinases (MAPKKKs, MEKK) and MAPK kinases (MAPKK, MEK) [27, 28]. Our study found that TNF-α inhibitors suppressed IL-17A , IL-17F and IL-22 production by decreasing p38 and ERK phosphorylation.

STAT3 and RORγt are lineage-specific transcription factors involved in Th17 differentiation. It has been reported that RORγt expression alone is insufficient to drive Th17 differentiation, and STAT3 activation augments the expression of RORγt during Th17 differentiation [15, 29]. The complete block of TNFα-induced STAT3 phosphorylation by p38-MAPK and ERK-MAPK inhibitors suggests a cross talk between the MAPK and STAT3 pathways [30]. In the present study, we found that TNF-α inhibitors suppressed pp38, pERK, and phospho-STAT3 protein expression 1 h after Th17 polarization, and RORγt mRNA expression was suppressed by TNF-α inhibitors 12 h after Th17 polarization. TNF-α inhibitors decreased IL-17 production through the downregulation of p38-MAPK, ERK-MAPK and STAT3 phosphorylation, which might subsequently suppress RORγt expression in Th17-polarized cells. These findings suggest a cross talk between the MAPKs and RORγt pathways.

An important novel finding in this study is the epigenetic regulation of the RORγt gene by TNF-α inhibitors. There is increasing experimental evidence of the pivotal role of epigenetic modifications in RA pathogenesis, but most studies have focused on the regulation of cytokine gene expression [19]. RORγt has been identified as a Th17-specific transcription factor that induces Th17 differentiation. In the present study, we found that anacardic acid, a nonselective inhibitor of HATs, suppressed RORγt expression in Th17-polarized cells. This finding suggests that histone acetylation is involved in RORγt expression. H3 and H4 acetylation, which commonly referred to as euchromatin modifications, are associated with active transcription [31]; therefore, we examined whether etanercept and adalimumab regulated RORγt expression through H3 and H4 acetylation in this study. To the best of our knowledge, this is the first study to investigate the epigenetic regulation of RORγt expression in Th17 cells. We found that TNF-α inhibitors could suppress RORγt protein and mRNA expression in human Th17-polarized cells, and these suppressive effects might be mediated through the downregulation of histone H3 and H4 acetylation. It has been reported that NFκB induction by T cell receptor stimulation enhances RORγt expression by binding to the RORγt promotor [32]. NFκB induces transcription after post-translational phosphorylation and acetylation of its subunits and then stimulates histone acetylation with p300, CBP and PCAF, which control gene transcription and have HAT activity [33]. In the present study, etanercept and adalimumab downregulated NFκB expression and decreased the recruitment of the acetyltransferases p300, CBP and PCAF to the RORγt promotor area. These findings suggest that TNF-α inhibitors inhibit the upstream transcription factor NFκB, which binds to the RORγt gene promoter and interacts with the coactivators CBP, p300 and PCAF, influencing the balance of HAT/HDAC activity and histone acetylation in the RORγt gene.

In addition to the important role of Th17 cells in the development of various autoimmune and inflammatory diseases, Th17 cells also provide host defence against bacterial and fungal infections [34]. Although TNF-α inhibitors exhibit significant clinical efficacy, they have many potential adverse effects, including reactivation of mycobacterial and fungal infection [35]. Previous studies have demonstrated that RORγt is required in mucocutaneous immunity to Candida and systemic immunity to Mycobacterium, and decreased pSTAT3 expression reduced IL-17 production in CD4^+^ T cells from tuberculosis patients [36, 37]. It has also been reported that p38-MAPK, NFκB and STAT3 play key roles in tissue destruction in patients with tuberculosis [38]. Our results on the suppression mechanisms of etanercept and adalimumab in Th17 polarization and cytokine production also provide additional understanding of the adverse effects of TNF-α inhibitors in reactivation of infection.

**Figure 8:**
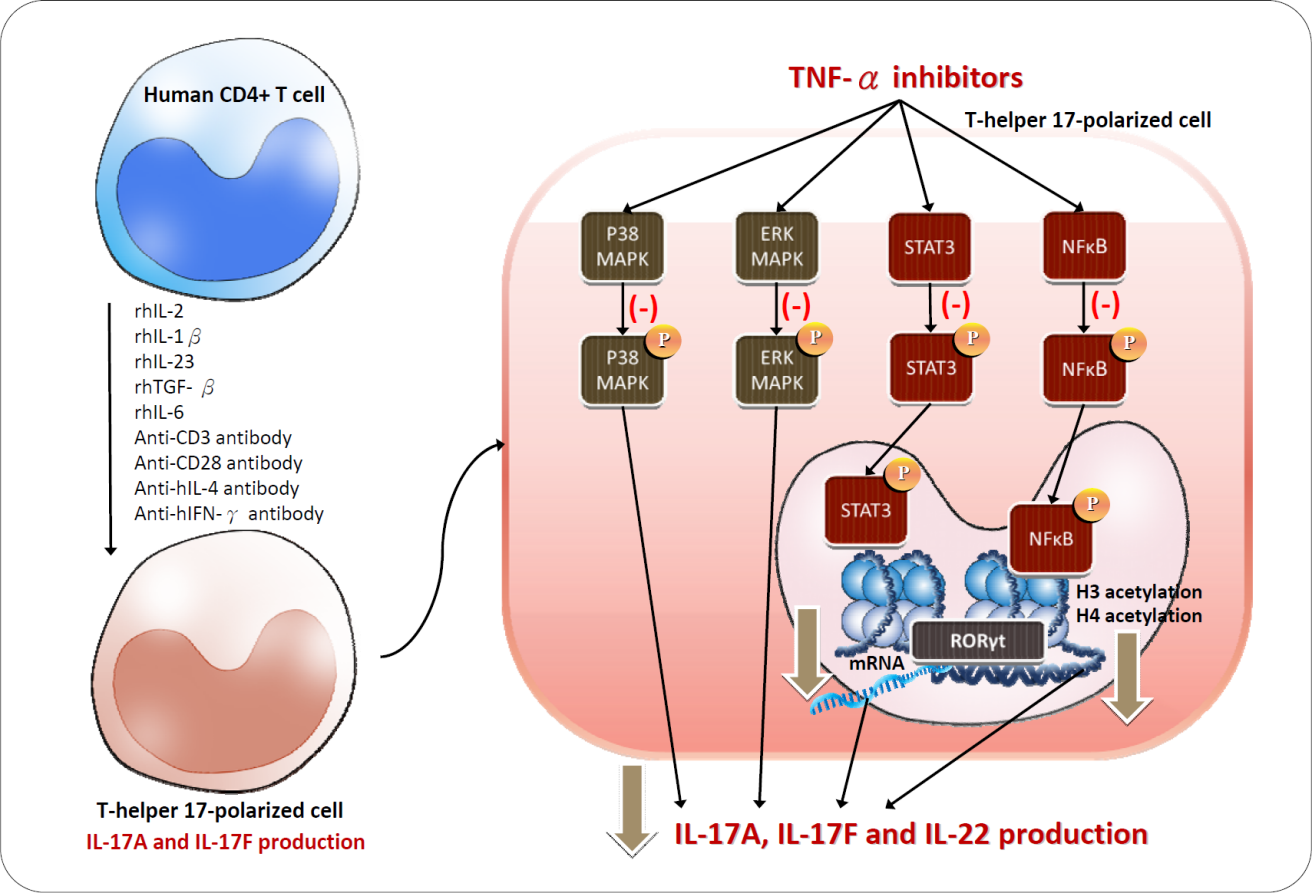
Schematic of the proposed intracellular mechanisms underlying TNF-α inhibitor regulation in Th17 cells polarized from CD4+ T cells

In conclusion, the present study provides further understanding of the TNF-α inhibitor-mediated suppression of the intracellular signalling pathways underlying Th17 differentiation and related cytokine production (Figure 8). Two common TNF-α inhibitors, etanercept and adalimumab, suppressed IL-17-related cytokine production through the p38-MAPK, ERK-MAPK and p65-NFκB pathways in Th17-polarized cells and downregulated phosphorylation of the linage-specific transcription factor STAT3 and histone acetylation at the RORγt gene. Some of the therapeutic efficacy of TNF-α inhibitors might result from the downregulation of these key transcription factors in Th17 cells. The present study established an experimental model of IL-17-producing cells polarized from human CD4^+^ cells and broadened our knowledge of the mechanism of TNF-α inhibitors in rheumatoid arthritis treatment.

## MATERIALS AND METHODS

### Purification and culture of CD4^+^ T cells

The study protocol was approved by the Institutional Review Board of Kaohsiung Medical University Hospital in Kaohsiung, Taiwan. Informed consent was obtained according to the Declaration of Helsinki. Four healthy subjects and six patients with RA were enrolled. Among these patients with RA, two were being treated with etanercept, three were being treated with adalimumab and one was being treated with metronidazole without any TNF-α inhibitors. PBMCs were isolated by density-gradient centrifugation (Lymphoprep™, Axis-Shield PoC AS, Oslo, Norway). Human CD4^+^ T cells were isolated from PBMCs by using microbeads (negative selection) according to the manufacturer's protocol (MACS, Miltenyi Biotec, Bergisch Gladbach, Germany). The purity of the CD4^+^ T cell population, which was analysed by flow cytometry, was more than 95%. Human CD4^+^ T cells were cultured in 24-well flat-bottomed plates at a density of 5 × 10^5^ cells/mL with anti-human CD3 mAb (1 μg/mL), anti-human CD28 mAb (1 μg/mL), and recombinant human (rh) IL-2 (5 ng/mL) in RPMI 1640 culture medium (Sigma Chemical Co., Saint Louis, Missouri, USA) supplemented with 10% foetal bovine serum, 100 U/mL penicillin, and 100 μg/mL streptomycin at 37°C with 5% CO_2_.

### Generation of Th17-polarized conditions from human CD4^+^ T cells

Th17-polarized conditions were generated by culturing human CD4^+^ T cells (5×10^5^/mL) with rhIL-2 (5 ng/mL), rhTGF-β (0.05 ng/mL), rhIL-6 (20 ng/mL), rhIL-1β (10 ng/mL), rhIL-23 (10 ng/mL), anti-hIL-4 mAb (1 mg/mL), anti-hIFN-γ mAb (1 mg/mL), anti-hCD3 mAb (1 mg/mL), and anti-hCD28 mAb (1 mg/mL) in a 24-well flat-bottomed plate in complete RPMI 1640 culture medium at 37°C with 5% CO_2_. Cells were pretreated with etanercept (Enbrel^®^; Immunex Corporation, Thousand Oaks, California, USA) or adalimumab (Humira^®^; AbbVie Incorporated, North Chicago, Illinois, USA) for 2 h and then stimulated with the Th17-polarized panel. To reduce pharmacokinetic variability between etanercept and adalimumab, the concentrations of these two TNF-α inhibitors were selected based on the therapeutic concentrations in the U.S. FDA-approved package inserts and clinical reports. A C_max_ of 1.1 ± 0.6 mcg/mL was observed in patients with RA following a single 25 mg dose [25]. In patients with RA receiving 40 mg of Humira^®^ every other week, adalimumab mean steady-state trough concentrations range from approximately 5 to 9 μg/mL [39]. Previous studies have reported that the cut-off trough levels of etanercept and adalimumab for a good therapeutic response in patients with RA are 1.046 to 1.56 μg/mL and 1.274 to 1.336 μg/mL, respectively [40, 41]. Therefore, etanercept was used at sub-therapeutic and therapeutic concentrations (0.1 and 1 μg/mL), and adalimumab was used at therapeutic and higher concentrations (1 and 10 μg/mL) in our study. The cell supernatants were collected after 5 days of Th17-polarized conditions.

To investigate cell signalling, the cells were pretreated with a p38-MAPK inhibitor (SB203580), JNK-MAPK inhibitor (SP600125) or ERK-MAPK inhibitor (PD98059) for 1 h before Th17 polarization. The concentration used in the experiments was based on the half-maximal inhibitory concentration (IC50) of each MAPK inhibitor and previous studies [42]. All the MAPK inhibitors were purchased from Cayman Chemical Company. Supernatants were collected for cytokine measurement.

### Enzyme-linked immunosorbent assay (ELISA)

Supernatant concentrations of IL-17A, IL-17F and IL-22 were measured with ELISA kits according to the manufacturer's protocol (R&D Systems Inc., Minneapolis, Minnesota, USA). The samples were measured with a Dynatech MRX plate reader at 450 and 540 nm using Revelation Software (Dynatech Laboratories Ltd., Virginia, USA).

### Cell viability

Th17-polarized cells were incubated with or without etanercept or adalimumab at various concentrations for 2 h prior to Th17 polarization. Cell viability after the 5-day Th17 polarization was examined using the CytoScan WST-1 Cell Proliferation Assay (G-Biosciences, Maryland Heights, MO, USA) according to the instructions of the manufacturer. The cell viabilities of each group were expressed as percentages of the control value.

### Western blotting

After treatment for 2 h with or without etanercept (0.1 and 1 μg/mL) or adalimumab (1 and 10 μg/mL), cells were stimulated with the Th17-polarized panel and subsequently lysed with an equal volume of ice-cold lysis buffer (150 μl) 1 h later for MAPKs, p65-NFκB and STAT3 signalling and 24 h later for RORγt signalling. After centrifugation at 13,000 × g for 15 min, cell lysates were analysed by western blot with anti-MAPK (p38 and ERK), anti-phospho-MAPK (pp38 and pERK), anti-p65, anti-phospho-p65, anti-STAT3, anti-phospho-STAT3, and anti-RORγt antibodies (Santa Cruz Biotechnology, Santa Cruz, California, USA). The immunoreactive bands were visualized using a horseradish peroxidase-conjugated secondary antibody and an enhanced chemiluminescence system (Amersham Pharmacia Biotech, Sunnyvale, California, USA).

### RNA extraction and real-time RT-PCR

Isolated human CD4^+^ T cells pretreated with etanercept (0.1 or 1 μg/mL) or adalimumab (1 or 10 μg/mL) for 2 h and were stimulated to become Th17-polarizing cells as described above. Total RNA was extracted from cells 12 h after Th17 polarization. RNA from each sample was reverse transcribed to first-strand cDNA using a SuperScript^TM^ First-Strand Synthesis System with an RT-PCR kit (Invitrogen, Carlsbad, California, USA). Measurements were made with an ABI PRISM 9700 HT sequence detection system (Applied Biosystems, Foster City, California, USA) using a TaqMan probe/primer combination for RORγt and glyceraldehyde 3-phosphate dehydrogenase (GAPDH) from the same cDNA samples. TaqMan PCR was performed using AmpliTaq Gold polymerase and Universal Master Mix (Applied Biosystems, Foster City, California, USA). Threshold cycle numbers were transformed using the comparative threshold cycle and relative value methods according to the manufacturer's recommendation and were expressed relative to GAPDH.

### Chromatin immunoprecipitation (ChIP) assay

ChIP was performed as described previously [42] with minor modifications. Antibodies against acetylated H3 and H4 as markers of gene activation [31] and antibodies against p300, CBP, and PCAF as NFκB-associated acetyltransferases were used [33]. Antibodies for the ChIP assay were purchased from Upstate Biotechnology Company. The primers were designed as previously described to analyse the promoter regions in the RORγt gene (forward: 5′-AGGCTGCACCACACTGG-3′, position: -599; reverse: 5′-TTCTACTCCTCCTACCCCCG-3′, position: -429) [43].

### Statistical analysis

Differences between the control and experimental groups were analysed using the Mann–Whitney *U* test. Differences in the intensities of the PCR-amplified products in the ChIP assay and the densitometry results in the western blot experiments between the control and experimental groups were analysed with Wilcoxon's signed-rank test. *P*-values less than 0.05 were considered significant.
